# Mesoporous bioactive glass combined with graphene oxide scaffolds for bone repair

**DOI:** 10.7150/ijbs.35670

**Published:** 2019-08-08

**Authors:** Wei Wang, Yang Liu, Chao Yang, Xin Qi, Shuangwu Li, Changsheng Liu, Xiaolin Li

**Affiliations:** 1 Department of Orthopedic Surgery, Shanghai Jiao Tong University Affiliated Sixth People's Hospital, Shanghai, China; 2 Engineering Research Center for Biomedical Materials of Ministry of Education, East China University of Science and Technology, Shanghai, China; 3 Department of Orthopedic Surgery, Shanghai General Hospital, Shanghai, China; 4 School of Engineering, King's College, University of Aberdeen, Scotland, United Kingdom

**Keywords:** oxide graphene, mesoporous bioactive glass, osteogenesis, angiogenesis, bone repair

## Abstract

Recently there has been an increasing interest in bioactive factors with robust osteogenic ability and angiogenesis function to repair bone defects. However, previously tested factors have not achieved satisfactory results due to low loading doses and a short protein half-life. Finding a validated stable substitute for these growth factors and apply it to the construction of porous scaffolds with the dual function of osteogenesis and angiogenesis is therefore vital for bone tissue regeneration engineering. Graphene oxide (GO) has attracted increasing attention due to its good biocompatibility, osteogenic, and angiogenic functions. This study aims to design a scaffold composed of mesoporous bioactive glasses (MBG) and GO to investigate whether the composite porous scaffold promotes local angiogenesis and bone healing. Our in vitro studies demonstrate that the MBG-GO scaffolds have better cytocompatibility and higher osteogenesis differentiation ability with rat bone marrow mesenchymal stem cells (rBMSCs) than the purely MBG scaffold. Moreover, MBG-GO scaffolds promote vascular ingrowth and, importantly, enhance bone repair at the defect site in a rat cranial defect model. The new bone was fully integrated not only with the periphery but also with the center of the scaffold. From these results, it is believed that the MBG-GO scaffolds possess excellent osteogenic-angiogenic properties which will make them appealing candidates for repairing bone defects. The novelty of this research is to provide a new material to treat bone defects in the clinic.

## Introduction

In recent years, methods for bone tissue engineering with the help of bioactive materials represent a promising strategy for bone repair after trauma, severe infection, tumor resection and congenital skeletal abnormalities 1, but it remains a major challenge in orthopedic surgery. Conventional surgical treatment has plateaued because the gold-standard autologous bone transplantation causes unavoidable secondary damage to the donor site 2, so implantation of a bioactive bone graft is needed to bridge the gap. The bone regeneration process needs close temporal and spatial coordination of events involving bone cells, marrow stroma and associated vascular elements 3. Neoangiogenesis, which is related to nutrient supply, is critical for bone repair 4. New bone formation and vascularization are often limited to the periphery of the scaffolds due to the damaged blood vessels in the center of the defect 4, 5. Methods that aim to address this issue have been explored, including the use of an expensive recombinant pro-angiogenic vascular endothelial growth factor (VEGF) and bone morphogenetic protein (BMP) in combination with tissue engineering scaffolds 6-8. These have strong properties of angiogenesis and osteogenesis, respectively. However, these approaches may prove problematic due to the high doses of proteins required, a short protein half-life and the inability to sustain biological activity easily 9, 10.

Finding a validated stable substitute for these growth factors and applying it to the construction of porous scaffolds with the dual function of osteogenesis and angiogenesis is a goal for bone tissue regeneration engineering.

Graphene oxide (GO), due to its high strength, large surface area and good cytocompatibility, has been widely investigated for various biomedical applications 11-15. GO's carboxyl, hydroxyl and epoxy groups promote interfacial interaction with polymeric matrices and ceramic, leading to their improved mechanical strength 16-18. GO is also capable of encouraging osteogenic differentiation and hydroxyapatite mineralization, thus increasing calcium fixation 19-22. Due to these properties, GO has been incorporated into several matrices aimed at bone regeneration, such as GO/TCP and GO/PLGA 23-26. Apart from the excellent biocompatibility, physical and osteogenesis properties of GO, recent studies have shown that graphene oxide can promote angiogenesis 27, 28. Angiogenesis is a basic process in bone tissue regeneration 4, 5, and GO can induce angiogenesis and contribute to nutrient formation and transportation in bone regeneration 29.

Another important aspect of bone tissue engineering is the three-dimensional scaffolds which provide a template for seeded cells to stimulate cell proliferation and differentiation, and also an interconnected pore structure to allow nutrients to penetrate into the scaffolds. Mesoporous bioactive glasses (MBG) have attracted increasing interest in bone tissue engineering in the last several years 30. The MBG scaffolds are similar to the porous structure of subchondral bone, because of their highly inter-connected large pores (300-500 μm) and ordered structure nanopores (2-50nm) 30. Consequently, MBG scaffolds promote greatly enhanced attachment, spreading and proliferation of cells, resulting in high bioactivity and degradation properties which benefit from the improved nanopore volume and surface area 31. However, the clinical translation of these scaffolds is impeded by weak osteogenic inducible activity for differentiation of osteogenic related precursor cells. As a solution, composite MBG scaffolds were developed to overcome the weak osteogenesis and angiogenesis by adding bioactive factors. It was difficult to achieve these two biological functions simultaneously by using only one bioactive growth factor due to the differing mechanisms. Recent research had reported that graphene improved cytocompatibility and significantly enhanced the hardness and Young's modulus of BGs 32. However, the use of low-dimensional material, such as GO, to enhance the performance of mesoporous bioglass to repair bone defects has not been reported.

In our study, we have explored a high temperature calcination technique to prepare MBG-based composite porous scaffolds using GO as an active reinforcer. We aim to determine whether these orderly porous scaffolds are suitable for cell adhesion and for promoting the proliferation, osteogenic differentiation of bone marrow mesenchymal stem cells and bone regeneration following a critical defect located in the cranium of rats.

## Materials and methods

### Materials

Graphene oxide with a lateral size of 0.8-1.2 nm and thickness of 0.5-5 µm were purchased from Nanjing XFNANO Materials Technology Co., Ltd (Nanjing, China). P123 (EO_20_PO_70_EO_20_) was obtained from Sigma-Aldrich (St Louis, MO, USA). Tetraethyl orthosilicate (TEOS), triethyl phosphate (TEP), and Ca(NO_3_)_2_•4H_2_O were obtained from Shanghai Ling Feng Chemical Reagent Co. Ltd. (Shanghai, China). All reagents were analytical grade and were used as received.

### Preparation of MBG-GO scaffolds

MBG was synthesized as previously described 31. Briefly, P123 (4.0 g), TEOS (6.7 g), Ca(NO_3_)_2_•4H_2_O (1.4 g), TEP (0.36 g; molar ratio of Si : Ca : P = 80 : 15 : 5), and 0.5 M HCl (1.0 g) were dissolved in ethanol (60 mL) and stirred at room temperature for 24 h continuously.

Porous MBG-GO scaffolds were prepared using a high temperature calcination technique. Briefly, GO was homogeneously mixed in a MBG solution and the ratios of GO : MBG were 0 : 4, 2.5 : 4, 25 : 4 (mg/mL). They were named MBG, MBG-LGO and MBG-HGO respectively. Each blend was mixed to form a homogeneous slurry and cast into a gelatin sponge with a diameter of 5 mm and a height of 2 mm. The gelatin sponge grouted by MBG-GO mixed solution was then dried at 60 °C. Finally, the dried scaffolds were calcined at 500 °C under nitrogen protection for 5 h to obtain the porous MBG-GO scaffolds. The scaffolds were sterilized using gamma irradiation.

### Characterization of scaffolds

The morphologies of the synthesized scaffolds were characterized by scanning electron microscopy (SEM, JEOLJSM-6701F) and high resolution micro-CT (mCT-80, Scanco Medical AG, Bassersdorf, Switzerland). The phase composition of the scaffolds were characterized by Fourier transformation infrared spectrum (FTIR, Bruker IFS66V FTIR spectrometer).

### *In vitro* cellular evaluation

Rat bone marrow mesenchymal stem cells (rBMSCs) were obtained from four femurs of Sprague-Dawley (SD) rats. Briefly, marrow of the femoral midshaft was extracted and then suspended in minimum essential medium alpha (α-MEM) containing 10% fetal bovine serum (FBS, Hyclone, Logan, UT, USA), 100 U/mL of penicillin and 100 mg/L of streptomycin (Hyclone). The non-adherent cells were discarded, and when the adherent cells reached 80-90% confluence they were passaged and became passage one (P1) cells. Sub-cultured rBMSCs at passages 4-5 were adopted in all* in vitro* cellular experiments.

### Cell attachment

To examine cellular morphology on the scaffolds, a 200 µL of rBMSC suspension containing 5×10^3^ cells was directly seeded onto the testing MBG and MBG-GO scaffolds. After 24 h of incubation, prior to SEM observation, the scaffolds were removed from the culture wells, rinsed with phosphate buffered saline (PBS) and fixed with 2.5% glutaraldehyde in PBS for 1 h. They were washed with PBS followed by sequential dehydration in graded ethanol (30% to 100%) and freeze drying. The cell-scaffolds were sputter-coated with gold and the morphological characteristics of the attached cells were characterized using SEM.

### Cell proliferation

The scaffold extracts were obtained following the International Standard Organization protocol (ISO 10993-5). Briefly, the scaffolds were immersed in minimum essential medium alpha (α-MEM) containing 10% fetal bovine serum (FBS, Hyclone, Logan, UT, USA), 100 U/mL of penicillin and 100 mg/L of streptomycin (Hyclone) in a cell incubator (humidified atmosphere with 5% CO_2_ at 37 °C) for 72 h (3cm^2^/mL). The supernatant was filtered and refrigerated at 4 °C for use within 7 days. The suspension containing 2×10^3^ cells was seeded into each well in a 96-well plate and the cells were incubated in humidified culture conditions. A Cell Counting Kit-8 assay (Dojindo Molecular Technologies, Inc. Japan) was performed to evaluate the cell proliferation of MBG and MBG-GO scaffold extracts. Briefly, 90 µL of culture medium and 10 µL of CCK-8 solution were added into each well at days 1, 3, and 7 and incubated at 37℃ for another 4 h. At the end of the incubation period, 100 µL of solution was removed from each well and transferred into another 96-well plate. The light absorbance was measured at 450 nm with a microplate reader (Bio-Rad 680, USA). All the results are presented as the optical density (OD) values minus the absorbance of blank wells. The study was performed in triplicate.

### ALP activity, staining and immunofluorescence evaluation

To evaluate the osteogenic effect, rBMSCs were cultured with the extracts for 7 and 14 days to study their osteogenic differentiation ability. At different time points, the cells were lysed using 100 µL RIPA lysis buffer, and the cell supernatant was collected into a 96-well plate. The alkaline phosphatase (ALP) activity in the supernatant was evaluated with the Alkaline Phosphatase Assay Kit (Beyotime, China). After co-incubation of extracts and p-nitrophenol for 30 mins at 37 ℃, the ALP activity was determined at a wavelength of 405 nm. Finally, the ALP levels were normalized to the total protein content determined by the bicinchoninic acid (BCA) Protein Assay Kit (Beyotime, China). The study was performed in triplicate. ALP staining was performed to detect ALP expression in the rBMSCs. At each predetermined time point, cells were washed with PBS three times, fixed with 4% paraformaldehyde for 15 mins, and incubated with the ALP staining kit (Beyotime) for 30 mins at 20 ℃ according to the manufacturer's protocol. After washing with PBS, the stained cells were examined using an inverted microscope (Leica DMI6000B, Solms, Germany). We also detected the ALP expression by immunofluorescence staining at day 7. Briefly, the cells were fixed by 4% paraformaldehyde for 15 mins and incubated in 0.1% Triton for 30 mins to permeabilise the cells. Non-specific protein-protein interactions of the cells were blocked by 1% BSA for 1 h. The cells were then incubated with the antibody ALP (1 : 200, Abcam108337) overnight at 4 ℃. The secondary antibody was donkey anti-rabbit Alexa Fluor 488 (1 : 200, Abcam) used for 1 h. Finally, the cytoskeleton and nuclei were stained with FITC-phalloidin and DAPI, respectively. A fluorescence microscope (Leica) was used to acquire representative images.

### Alizarin red S staining and OCN immunofluorescence assay

rBMSCs were cultured as described above. At day 14, the cell layers were fixed with 4% paraformaldehyde for 15 mins, and washed with PBS three times followed by adding 2% Alizarin red S solution (Cyagen) for 10 mins. Cells were washed with PBS three times and the mineralized nodules were then examined using an inverted microscope (Leica DMI6000B, Solms, Germany). Finally, the staining was extracted by adding 10% cetylpyridinium chloride (Sigma-Aldrich Co., USA) for 15 mins at 37 ℃. The absorbance was recorded at a wavelength of 595 nm using a microplate reader (Bio-Rad 680, USA). We determined a later osteogenic differentiation protein, osteocalcin (OCN), by immunofluorescence staining at day 14. Briefly, the cells were fixed in 4% paraformaldehyde for 15 mins, permeabilized by 0.1% Triton-X for 30 mins, blocked using 1% BSA for 1 h and incubated with primary antibodies for OCN (1 : 200, Abcam13420) overnight at 4 ℃. Secondary donkey anti-mouse Alexa Fluor 488 (1 : 200, Abcam) was applied to combine with the primary antibody for 1 h. Finally, the cytoskeleton and nuclei were stained red and blue with FITC-Phalloidin and DAPI, and observed with a fluorescence microscope (Leica).

### Osteogenesis/Angiogenesis related genes expression of rBMSCs

The expression levels of osteogenesis/angiogenesis-related genes (alkaline phosphatase (ALP), runt-related transcription factor 2 (RUNX-2), osteocalcin (OCN), collagen type 1 (COL1)), vascular endothelial growth factor (VEGF) and hypoxia-inducible factor 1-alpha (HIF-1α) were measured using quantitative reverse transcription polymerase chain reaction (qRT-PCR) analysis. Typically, the cells were seeded at a density of 2 × 10^4^ cells per well, cultured for 7 days in a 6-well plate, then harvested using Trizol Reagent (Invitrogen Carlsbad, CA, USA) to extract the RNA. The obtained RNA was reverse-transcribed into complementary DNA (cDNA) using a Revert Aid First Strand cDNA Synthesis Kit (Thermo Fisher Scientific, Waltham, MA, USA) and qRT-PCR analysis was performed on an ABI Prism 7300 Thermal Cycler (Applied Biosystems, Foster City, CA, USA) using SYBR Green detection reagent. GAPDH was employed as the housekeeping gene for internal normalization. All samples were assayed in triplicate and independent experiments were performed three times. The relative expression was calculated using the formula: 2^-△△Ct^. Primer information is given in Table 1.

### Cranial bone defect model and artificial scaffolds implantation

The SD rat cranial bone defect model was used to investigate the osteogenic capacity of the scaffolds in vivo. The experimental procedures, housing and animal care were approved and carried out in accordance with the regulations for animal experiments of the Animal Ethics Committee of Shanghai Sixth People's Hospital-affiliated Shanghai Jiao Tong University. Eight-week-old male SD rats were obtained from Shanghai Xipuer-Bikai Laboratory Animal Co., Ltd (Shanghai, China) and housed in a standard SPF animal laboratory. After adaptation for one week, 250-300g SD rats were used for establishing the critical cranial bone defect model. For the surgical procedure as previously described 33, the cranium was exposed through a central incision after general anesthesia with an intraperitoneal injection of 0.5% pentobarbital sodium (9 mL/kg body weight). Two critical-sized calvarial defects with a diameter of 5 mm were created on each side of the cranium using a dental trephine irrigated by ice saline solution to avoid thermal injury. After the bone was removed, the drilled holes were rinsed with saline solution and the 5 mm × 2 mm scaffolds were then randomly implanted into the defects. Following the operation, the animals received intramuscular antibiotic injections, were allowed free access to food and water and were monitored daily for potential complications. In total, 24 animals were divided into three groups as follows: (1) MBG scaffolds group, n = 8 (2) MBG-LGO scaffolds group, n = 8 and (3) MBG-HGO scaffolds group, n = 8.

### Microcomputed tomography (micro-CT)

To evaluate the in vivo bone ingrowth of the implanted porous scaffolds, craniums were harvested and evaluated at 12 weeks using a high-resolution micro-CT (mCT-80, Scanco Medical AG, Bassersdorf, Switzerland) at an isometric resolution of 18 μm. Scanco software was used for analysis. Three-dimensional grayscale images were generated using the CTVol program. As there are density differences between scaffolds and new bone, CTAn software used in this study can differentiate between them. Percentage of new bone volume relative to tissue volume (BV/TV) and bone mineral density (BMD) in the bone defect were both calculated.

### Microfil perfusion in the bone defect

The vasculature of SD rats was injected with 20 mL of silicone rubber compound (Microfil MV-122, Flow Tech, Carver, MA) after they were euthanized at 12 weeks post-operation 34. Briefly, the animals were anesthetized and the rib cage was opened. The descending aorta was clamped and the auricula dextra was incised. Heparinized saline and Microfil were successively perfused into the left ventricle with an angiocatheter. Successful perfusion was defined as a yellow color change in the eyes and tongue. Finally, the rats were stored at 4 °C overnight to ensure plasticization of the contrast medium, after which the crania were dissected and fixed in 4% paraformaldehyde for another 48 h. The fixed crania were decalcified in 10% ethylenediaminetetraacetic acid (EDTA; Sigma, US) for four weeks. Images were obtained with a high-resolution micro-CT imaging system at 9 µm resolution, and the number and volume of vessels within the 5 mm diameter region surrounding the bone defect were evaluated.

### Sequential fluorescent labeling in the bone defect

At 2, 4, and 6 weeks after the operation, the SD rats were intraperitoneally injected with tetracycline (TE, 25 mg/kg of body weight), alizarin red (AL, 30 mg/kg of body weight) and calcein (CA, 20 mg/kg of body weight). The mineralized tissue was observed using the trichromatic sequential fluorescent labeling method 3.

### Newly bone formation and mineralization analysis

One part of each specimen was dehydrated in ascending concentrations of alcohols from 70% to 100% and embedded in polymethylmethacrylate (PMMA). After hardening, the sagittal sections of the specimens were cut into 150 μm thick slices using a microtome (Leica Microsystems Ltd., Wetzlar, Germany), followed by grinding and polishing to a final thickness of approximately 50 μm. The sections were first viewed using confocal laser scanning microscopy (CLSM) (Leica) to examine fluorescent labeling. New bone formation and mineralization were quantified at six locations of the defect site. The mean value of the six measurements was calculated to obtain average values for each group. The sections were then stained with van Gieson's staining to identify new bone formation. Red indicated new bone formation, and black indicated residual materials 35. The area of new bone formation was evaluated quantitatively in six randomly-selected sections using Image Pro 5.0 (Media Cybernetics, Rockville, MD, USA).

### Immunohistochemical (IHC) analysis

The other half of the craniums was decalcified for 4 weeks in 10% EDTA solution, dehydrated with gradient alcohols, embedded in paraffin, and then sectioned into 4 µm thickness sections. Osteogenesis and angiogenesis were evaluated by IHC analysis for osteocalcin (OCN, abcam13420) and CD34 (abcam81289).

### Statistical analysis

All the above data are presented as mean ± standard deviation (SD). Differences between groups were calculated by one-way analysis of variance (ANOVA) and Student-Newman-Keuls post-hoc tests. The statistical analysis was conducted using SPSS 17.0 software (SPSS Inc., Chicago, IL, USA). The difference was considered significant when *P* < 0.05.

## Results

### Characterization of MBG and MBG-GO composite scaffolds

**Fig 2:**
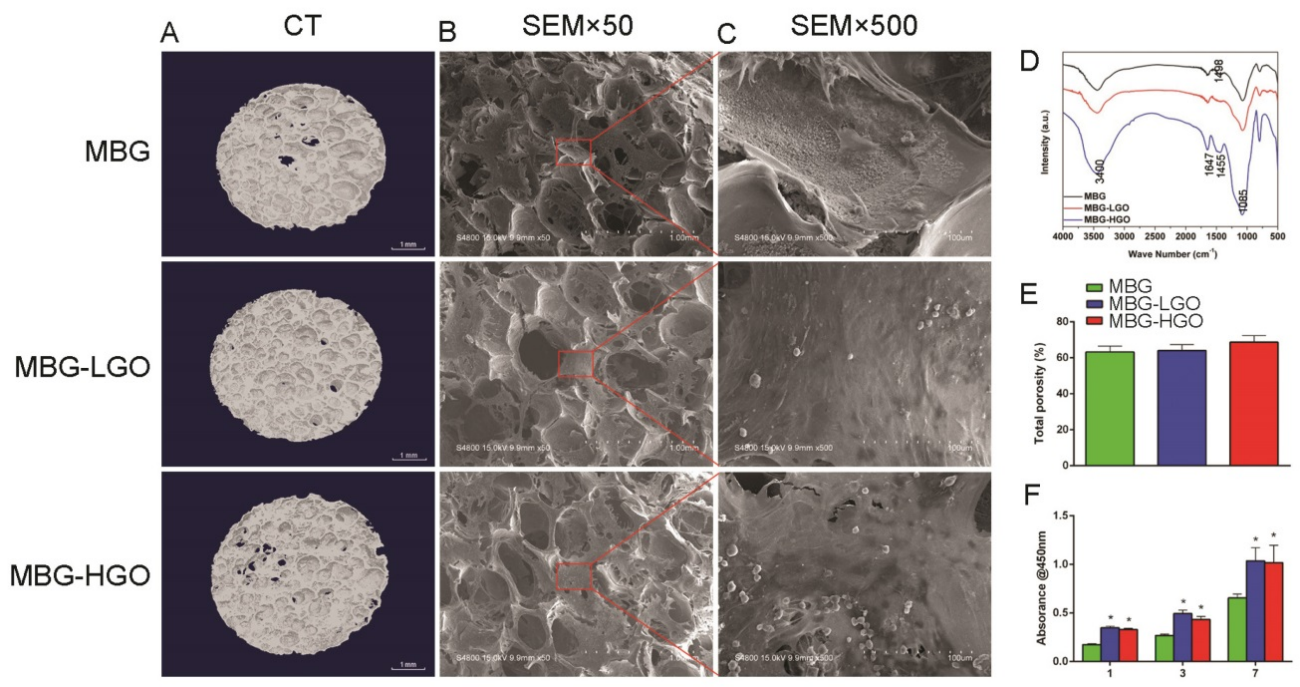
(A) 3D images of scaffolds by Micro-CT. (B-C) The pore size and cell adhesion of scaffold by SEM. (D) The phase composition of the scaffolds were characterized by FTIR. (E) Quantitative analysis of total porosity. (F) The proliferation of rBMSCs cultured with scaffolds extracts. (* represent *p* < 0.05 when compared with MBG).

To verify that MBG was successfully bound with GO through a chemical bond combination, FTIR spectra were obtained (Fig. 2D). For MBG and MBG-GO, both broad bands appeared at around 3400 cm^-1^, which was associated with the O-H stretching vibration of adsorbed water molecules. After binding with MBG, the typical carbonyl group of GO disappeared at 1730 cm^-1^. A new band of composites appeared at 1085 cm^-1^, which was attributed to the (Si-O-C/Si-O-Si) asymmetric stretching vibration. This evidence proved that the carbonyl groups were converted to Si-O-C bands. For pristine MBG, the band at 1498 cm^-1^ conformed to the band range of Ca-O phase at 1450-1700 cm^-1^, confirming the existence of a Ca-O phase in the structure. SiO_2_ characteristic bands also appeared in the spectrum of MBG. The existences of Ca-O phase and Si-O phase demonstrated that the synthesized MBG are composed of CaO and SiO_2_. Among the MBG-GO composites, the characteristic bands of MBG appeared on the spectrum in addition to those of GO.

To observe the pore size and porosity of the scaffolds, SEM and micro-CT scans of the materials were performed. The results show that the three types of scaffolds have similar pore sizes (300-500 µm) and porosity (63.2%-68.7%) (Fig. 2A-B, 2E). It is observed that the content of GO had no significant effect on the pore diameter and porosity of the scaffold, even increasing the porosity of the scaffold to some extent.

### Biocompatibility of the scaffolds with rBMSCs

rBMSCs were cultured on scaffolds to investigate the cell compatibility of porous MBG-GO scaffolds. The attachment and morphology of cells on scaffolds were observed by SEM (Fig. 2B-C). After being cultured for 24 h, rBMSCs attached to the surface of the pore struts in scaffolds. Well-spread morphology was observed and the pore walls of MBG-GO groups were almost completely covered by cytoskeleton. As determined by a CCK-8 proliferation assay (Fig. 2F), all MBG and MBG-GO scaffold extracts supported cell proliferation well. However, the proliferation rates of MBG-GO scaffolds were significantly higher than those of MBG group at days 1, 3 and 7 (*P* < 0.05). The MBG-LGO group showed the best rate, but the difference was not statistically significant when compared to the MBG-HGO (*P* > 0.05).

### Osteogenic differentiation effect of rBMSCs cultured with extracts

**Fig 3:**
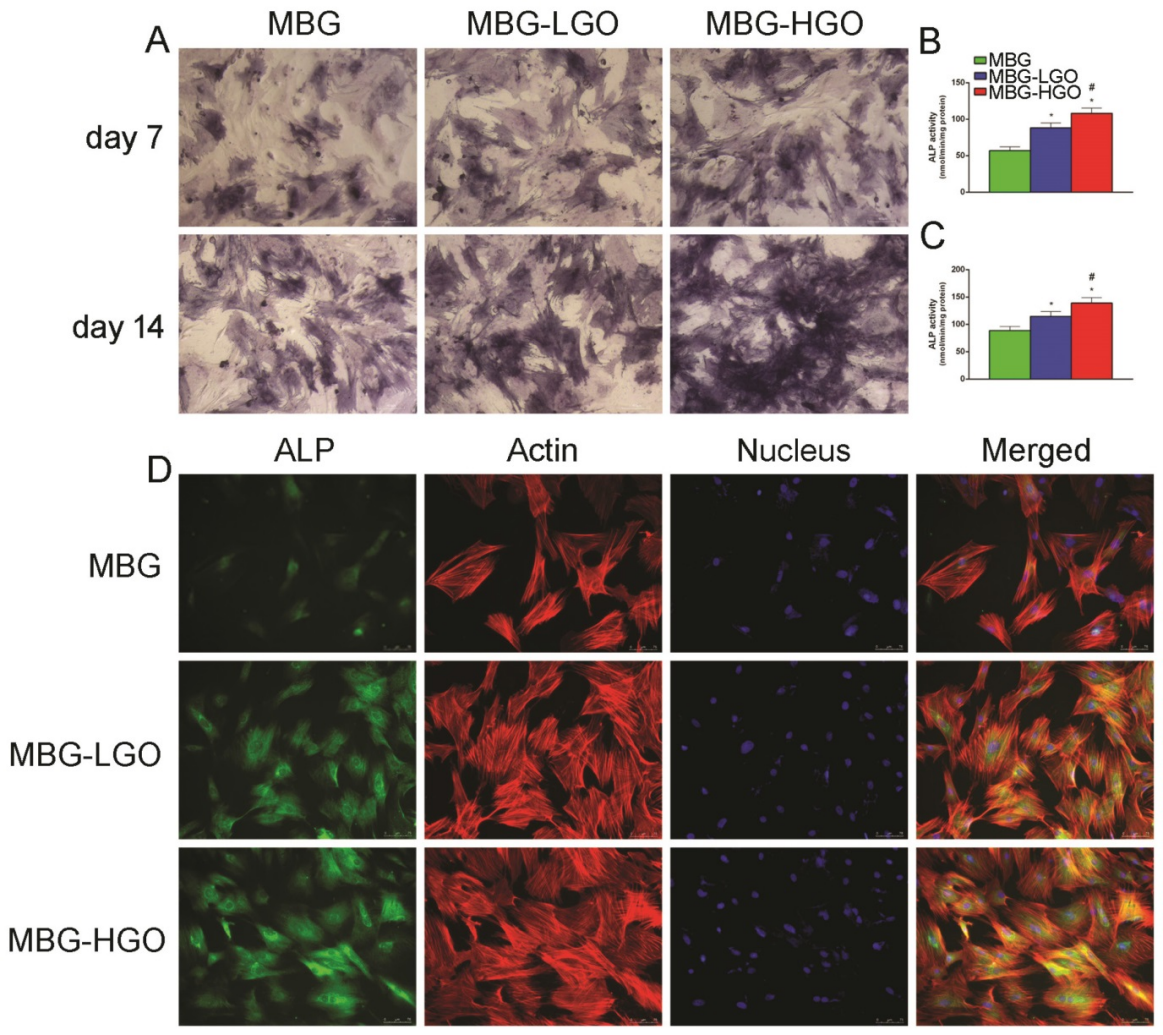
(A) ALP staining of rBMSC cultured with scaffolds extracts for 7 and 14 d. (B-C) Quantitative analysis of ALP activity. (* and # represent *p* < 0.05 when compared with MBG, MBG-LGO, respectively). (D) ALP immunofluorescent staining of rBMSC cultured with scaffolds extracts for 7 d: green (ALP), red (actin), blue (nucleus).

Fig. 3A reveals that ALP expression increased over time, and the highest ALP expression was observed in the MBG-HGO group, followed by the MBG-LGO group. Consistent with the ALP staining results, a similar trend was observed in ALP activity and immunofluorescence staining assays, with the highest ALP activity and ALP (green) fluorescence intensity detected in the MBG-HGO group (Fig. 3B-D).

**Fig 4:**
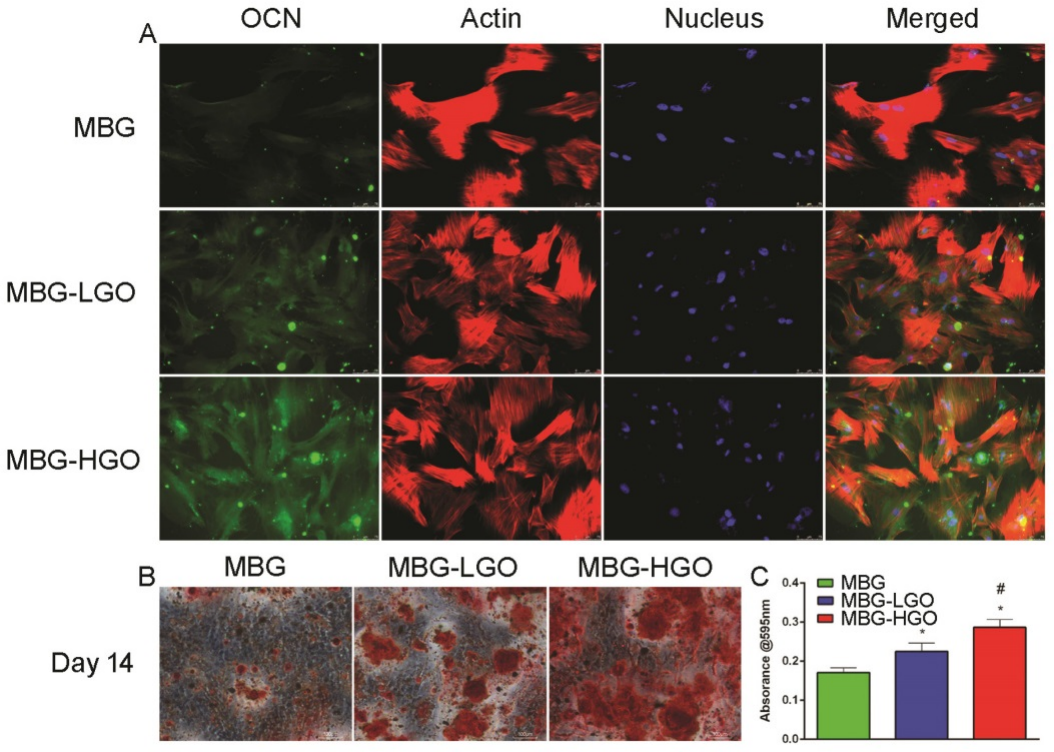
(A) OCN immunofluorescent staining of rBMSC cultured with scaffolds extracts for 14 d: green (OCN), red (actin), blue (nucleus). (B) Alizarin Red staining of rBMSC cultured with scaffolds extracts for 14 d. (C) Quantitative analysis of Alizarin Red staining. (* and # represent *p* < 0.05 when compared with MBG, MBG-LGO, respectively).

A later osteogenic differentiation protein, osteocalcin (OCN), was detected by immunofluorescence staining at day 14. The results showed that rBMSCs cultured with the MBG-HGO extract expressed more OCN (green) than those in the MBG or MBG-LGO group (Fig. 4A). To study the mineralization level of rBMSCs cultured with scaffold extracts, Alizarin red S staining was conducted at day 14. A great number of calcified nodules were stained red in the MBG-HGO group than in the other groups (Fig. 4B), and the trend was further confirmed by the quantitative test shown in Fig. 4C.

**Fig 5:**
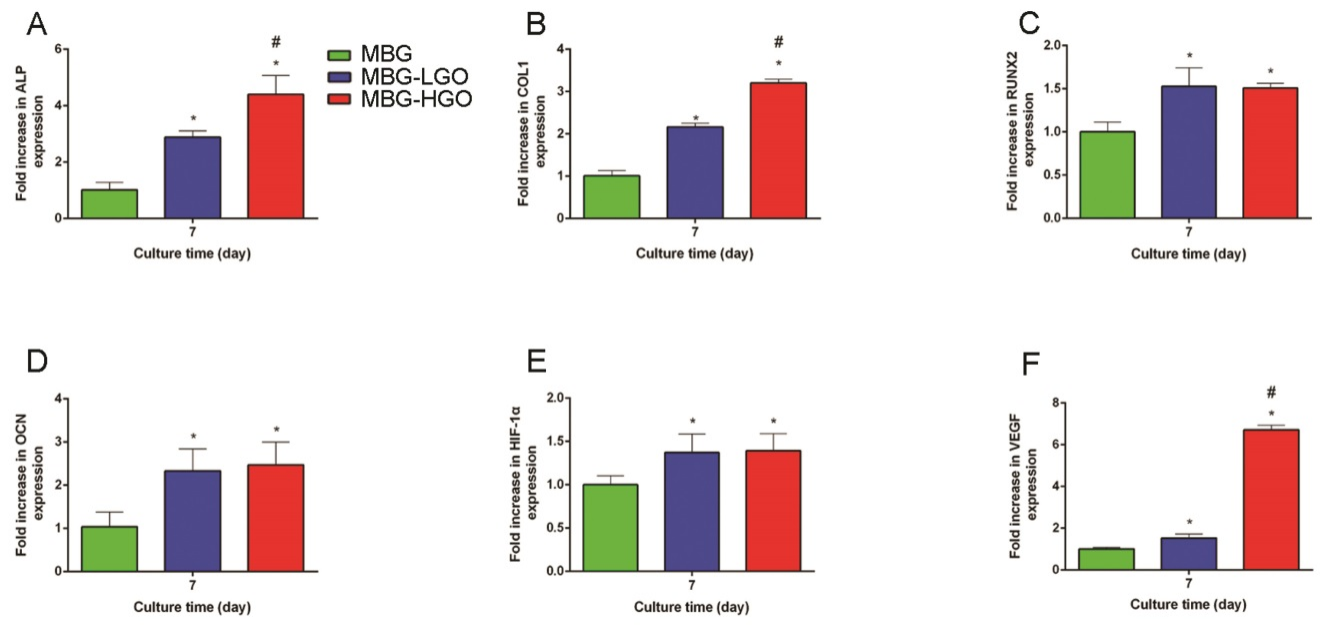
Gene expression analysis of rBMSCs cultured with scaffolds extracts. (A-F) qRT-PCR results of ALP, COL1, RUNX2, OCN, HIF-1α, and VEGF respectively. (* and # represent *p* < 0.05 when compared with MBG, MBG-LGO, respectively).

To further clarify the osteogenic differentiation effect of rBMSCs cultured with scaffold extracts, several marker genes which were essential during osteogenesis were examined. The results showed that these osteogenic-related genes were all up-regulated in cells cultured with MBG-GO extracts compared to the MBG group (*P <* 0.05) (Fig. 5A-D). Simultaneously, we found that the expressions of the ALP and COL1 genes were stronger in the MBG-HGO group than in the MBG-LGO group (*P <* 0.05) (Fig. 5A-B), indicating that the MBG-HGO scaffold extract could enhance osteogenic differentiation better.

### Porous MBG-GO scaffolds promotes bone regeneration in vivo

**Fig 6:**
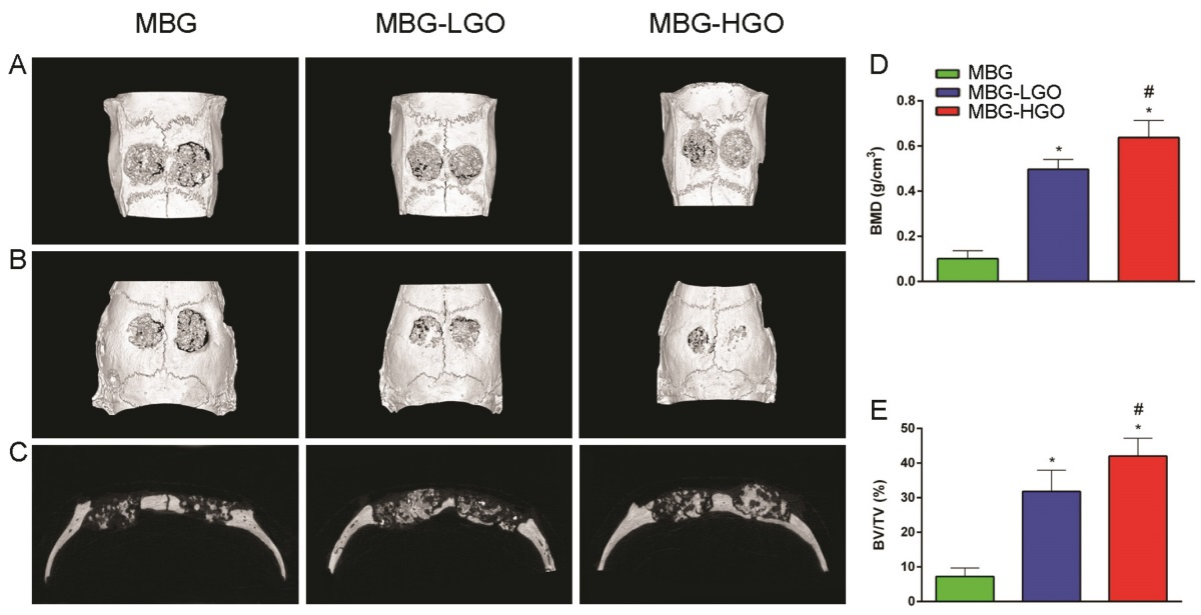
Results of the in vivo bone-repairing model. (A-B) 3D images on front and back of Micro-CT. (C) Sagittal images of Micro-CT. (D-E) Quantitative analysis of Micro-CT data: BMD(g/cm^2^), BV/TV(%) respectively. (* and # represent *p* < 0.05 when compared with MBG, MBG-LGO, respectively).

According to the results of in vitro study, we further studied the in vivo osteogenesis effect of porous MBG-GO scaffolds with a large cranial defect in rats. The rats survived well; none of them died or developed infections during the course of the study after scaffold implantation. Three-dimensional micro-CT reconstructed images showed the morphology of the newly-formed bone (Fig. 6A-B). In the sagittal plane (Fig. 6C), more newly-formed bone was observed in MBG-HGO scaffold group than in other groups. Quantitative analysis of the newly-formed bone was performed by the image analysis system. The local BMD was markedly higher in the MBG-HGO scaffold group (0.64 ± 0.08 g/cm^3^) than that in the MBG scaffold group (0.10 ± 0.04 g/cm^3^), or in the MBG-LGO scaffold group (0.50 ± 0.04 g/cm^3^) (*P <* 0.05) (Fig. 6D). The differences in BV/TV between these groups also showed the same pattern (Fig. 6E). The results indicate that MBG-GO scaffolds can significantly improve bone regeneration and that BMD increases with the increasing content of GO.

### Porous MBG-GO scaffolds promote vascularization in vivo

**Fig 7:**
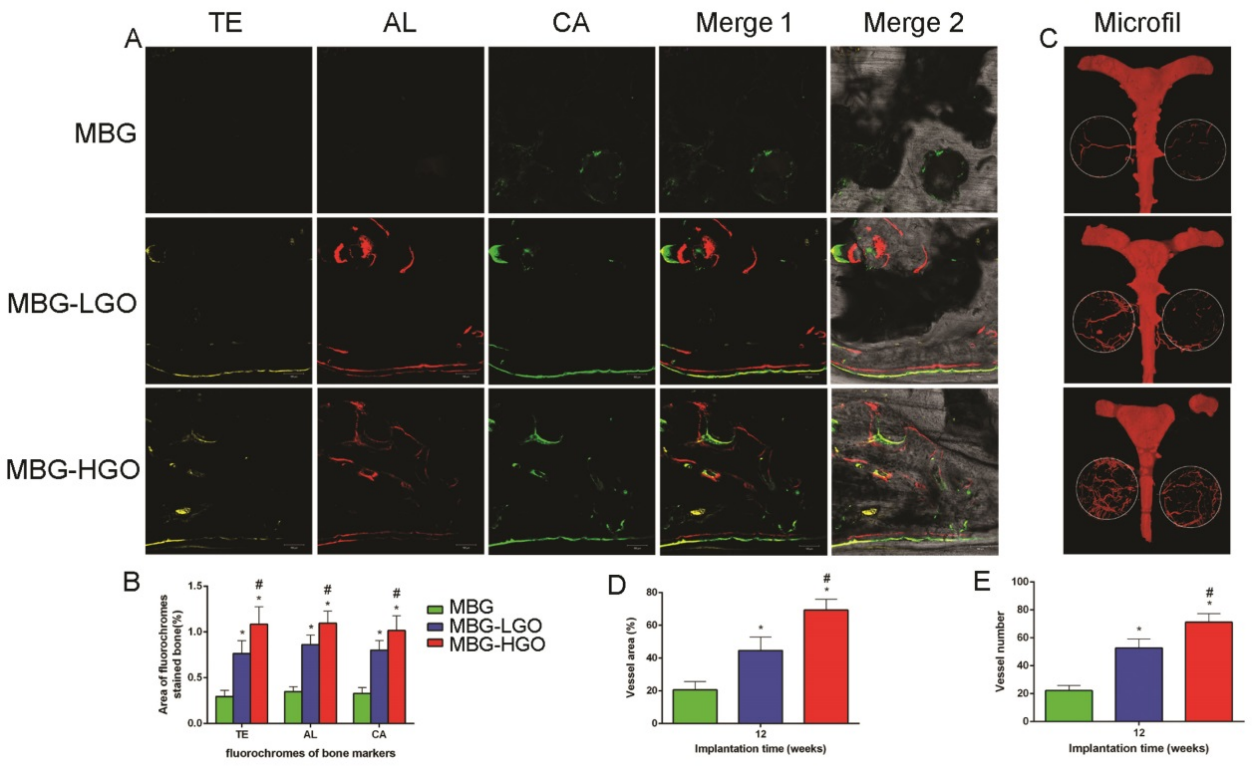
(A) New bone formation and mineralization of scaffolds by sequential polychrome labels analysis: yellow (Tetracycline), red (Alizarin red), green (Calcein). (B) Quantitative analysis of sequential polychrome labels. (C) Microfil evaluation of neovascularization in the defect area (white circles indicated). (D-E) Quantitative analysis of neovessels: vessel areas, vessel number, respectively. (* and # represent *p* < 0.05 when compared with MBG, MBG-LGO, respectively).

In our in vitro study, we studied two marker genes, VEGF and HIF-1α, which were essential during angiogenesis. The results showed that these were both significantly up-regulated in MBG-GO groups compared to the MBG group when cultured with scaffold extract for 7 days (Fig. 5E-F). To clarify the effects of local vessel formation in scaffolds after 12 weeks of implantation, micro-CT imaging was carried out. Three-dimensional reconstruction images were obtained and typical images were displayed (Fig. 7C). We could observe the newly formed vascular networks in the defect area from the corresponding images. They showed that the MBG group had almost no new visible vascular formation, whereas in the other groups a considerable number of vessels extended along the scaffolds from the edge of the defects. The number and of new vessels in the MBG-HGO group were both larger than for the other groups (Fig. 7D-E). Similar to of new bone growth, large groups of vascular networks also existed in the center of the MBG-HGO group (Fig. 7C).

### Newly bone formation and mineralization analysis

As shown in Fig. 7A, new bone formation and mineralization were analyzed at 2, 4 and 6 weeks by sequential fluorescence labels. At 2 weeks, the percentage of TE labeling (yellow) in the MBG-HGO scaffold group (1.09 ± 0.19%) was greater than that in the MBG scaffold group (0.29 ± 0.07%), or the MBG-LGO scaffold group (0.76 ± 0.14%) (*P* < 0.05). At 4 weeks, the highest percentage of AL labeling (red) was observed in the MBG-HGO scaffold group (1.09 ± 0.13%), but there was also a significant difference between the MBG-LGO scaffold group (0.86 ± 0.10%) and the MBG scaffold (0.35 ± 0.05%) (*P* < 0.05). At 6 weeks, the percentage of CA labeling (green) in the MBG-HGO scaffold group (1.02 ± 0.16%) was significantly higher than that in the MBG scaffold (0.33 ± 0.06%), or in the MBG-LGO scaffold group (0.80 ± 0.11%) (*P <* 0.05) (Fig. 7B). The results indicate that GO can promote bone formation at early stages.

**Fig 8:**
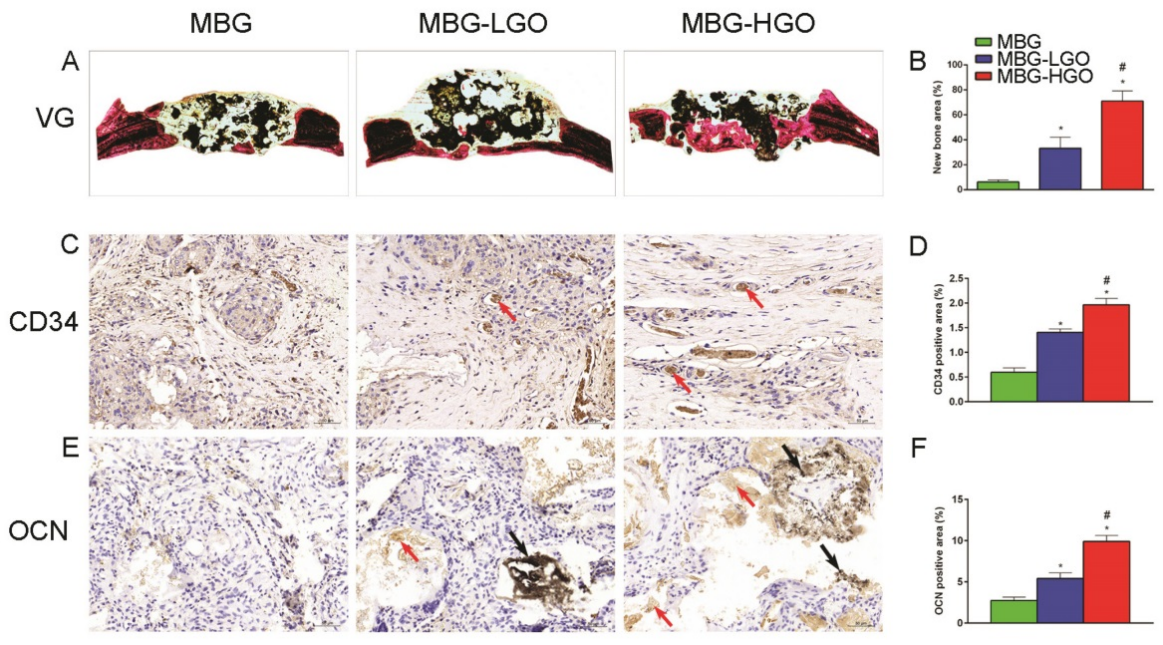
(A) Van Gieson staining of undecalcified sections, red indicates bone tissue and black indicates the residual scaffold material in defect site. (C, E) Histological analysis of decalcified sections: red arrow indicates new vessels and new bone, black arrow indicates graphene oxide. (B, D, F) Quantitative analysis of newly bone tussues, neovessel and OCN. (* and # represent *p* < 0.05 when compared with MBG, MBG-LGO, respectively).

Consistent with the above results, histological analysis using van Gieson staining of undecalcified specimens showed extensive new formation of bone in the defect areas (Fig. 8A). Bone regeneration was markedly increased in the MBG-HGO scaffold group (71.05 ± 8.07%), with the new bone formation area significantly greater than that in the MBG scaffold (6.17 ± 1.59%), and in the MBG-LGO scaffold groups (33.28 ± 8.97%) (*P <* 0.05) (Fig. 8B).

### Immunohistochemical (IHC) analysis

To further clarify the osteogenic and angiogenic functions of stents in vivo, the osteogenic and angiogenic markers OCN and CD34 were detected by immunohistochemical staining of decalcified cranial specimens. There was virtually no obvious positive staining for OCN/CD34 in the pury MBG scaffold group, but positive brown staining for OCN/CD34 was apparent in the MBG-GO groups (Fig. 8C, E), and greater positive staining was found in the MBG-HGO scaffold group (Fig. 8D, F). The analysis of bone regeneration in cranial defects indicated that MBG-GO scaffolds can significantly improve newly bone formation and neovascularization, which increased in line with the increasing content of GO. Results were consistent with the previous micro-CT results.

## Discussion

The regeneration process of bone defects primarily relies on a physical bridge between defect ends and the chemical guidance of bioactive molecules and proteins. Bioactive ceramics have been widely accepted and used as a successful biomaterial in studies of bone repair and drug carriers 1-3, 6, 30, 31, 36-39. Particularly in the bone tissue engineering field, various bioactive factors like DMOG, VEGF and BMP-2 encapsulated in mesoporous bioactive glasses have already been tested to enhance the function of osteogenesis and angiogenesis 3, 6-8. However, it is difficult to achieve these two biological functions simultaneously by only using one bioactive growth factor due to the differing mechanisms. Their inherent shortcomings, including short half-life, low activity, side effects at larger physical dosage and potential immune reaction, severely restrict their application in clinical settings 9, 10. To achieve fully functional and structural recovery, an advanced bioactive scaffold is needed to provide an ideal environment for bone tissue regrowth. In recent years, low-dimensional nano-materials including carbon nanotubes, graphene, and boron nitride nanotubes have shown significant potential in reinforcing bioactive ceramics because of their unique structures and properties 40. GO is a representative new conductive material with the ability to enhance mechanical properties, cell attachment, proliferation and more importantly, osteogenesis-angiogenesis 16, 22, 29, 41. Thus, most studies on graphene oxide composites have focused on polymer matrices. Few studies have been carried out on graphene oxide/glass ceramic composites. Considering that skeletal development occurs in close spatial and temporal association with angiogenesis 3, in this study MBG was responsible for carrying GO to enhance osteogenesis and angiogenesis.

GO is a nano-sized particle with excellent dispersion properties in water and ethanol 42, and thus GO can be mixed into a MBG solution homogeneously. In addition, comparing with other metal elements, such as copper and cobalt, which are beneficial to bone formation 43, 44. The chemical structure of GO contains an abundance of hydroxyl, epoxy and carboxyl groups on the basal planes of the GO sheets, and GO therefore possesses large surface area and enhances cells adhesion 16. It can therefore react with TEOS to ensure that the MBG can firmly bind with GO sheets 32, 45. In this study, we varied the weight ratio of MBG to GO to synthesize three types of composite scaffolds, consisting of purely MBG, MBG-LGO and MBG-HGO. By using a high temperature calcination technique, the scaffold can be produced with symmetrical macroporous struts and mesoporous interfaces. We also confirmed that MBGs can firmly bind with GO sheets via FTIR assay. The porosity of scaffolds increased slightly with the addition of graphene oxide. The pore size of the MBG-GO scaffolds (300-500 μm) permits the free exchange of nutrients, such as necessary proteins, oxygen and water, thus facilitating the delivery of energy for bone regeneration and the formation of capillaries 46. In this study, we also confirmed that the size of 300-500 μm is conducive to cell adhesion and the growth of new bone and blood vessels.

Excellent cytocompatibility and osseointegration with the host are a prerequisite for biomaterials. These are important criteria in evaluating whether a biomaterial can be implanted in vivo 32. The surface physicochemical properties of scaffolds are important for cell behavior. A succession of processes occur in the initial adhesion of cells with implants. Cell adhesion directly impacts cell growth, migration, and differentiation. Direct cellular adhesion and subsequent cellular responses are therefore critical and prerequisite parameters for osteointegration and osteoconduction 47, 48. Herein, the cytocompatibility of MBG-GO composites were first detected by SEM assay, which quantifies cellular activity when exposed to materials and is a commonly used method to analyze the possible harmful effects that materials induce in cells. When the cells were cultured for 24 h, the cells on the surface of MBG, MBG-LGO and MBG-HGO scaffolds all maintained their fusiform shapes, but the cells spread out more on the surfaces of MBG-LGO and MBG-HGO scaffolds. This result further demonstrated the functional bioactive environment provided by GO. The viability and proliferation of these cells were assessed by CCK-8 test after one, three, and seven days of incubation. According to the data shown in Fig. 2F, MBG-GO groups had a significantly higher cell viability rate than that of the control group, indicating that GO has a significant effect on the proliferation of the rBMSC cells. The enhanced proliferation was further demonstrated by immunofluorescence assays in which more cytoskeletons and nuclei were seen at high magnification (Fig. 3A, 4A). The results from SEM and CCK-8 assays indicated that a small amount of GO had no obvious cell toxicity, but excessive GO content may inhibit cell proliferation due to toxicity. Differentiation of rBMSCs is the key process for bone regeneration. It has been demonstrated that GO can enhance the osteogenic activity of osteoprogenitor cells and stimulate in vivo bone regeneration 19, 26. In our study, we also confirmed that the binding of GO significantly promoted rBMSC osteogenic differentiation and new bone formation. Generally, the differentiation of cells are the significant steps that occur before bone mineralization. The fundamental processes of cell differentiation and function are governed by the interaction of cells with their substrate 31, 49. GO could promote osteogenic differentiation through activation of the Wnt/β-catenin signalling pathway, and the effect of osteogenesis is seen to be concentration-dependent 50. In our vitro study, ALP, RUNX2, OCN and COL1 gene secretion were significantly enhanced in MBG-GO groups, and the results of qRT-PCR were further confirmed by ALP staining, mineralized nodules staining, and ALP/OCN cells immunofluorescence tests. The comprehensive use of micro-CT, histological examination fluorochrome labelling and IHC revealed that more intensive bone formation was detected in the MBG-GO group, which indicates that GO stimulated the participation of rBMSCs in the repair of bone defects.

Microvessels are also vital to bone regeneration 4, 5. To verify the influence of the different scaffolds on angiogenesis in the process of bone regeneration, various markers were used including VEGF, HIF-1α and CD34. The HIF-1 complex, one of the most important angiogenesis signalling pathways, initiates numerous gene expressions including VEGF, and modulates stem cell proliferation, differentiation and pluripotency 51. CD34, which belongs to a family of single-pass transmembrane proteins, is closely associated with vascular-associated tissue 52. Low concentrations of GO can promote the expression of VEGF and angiogenisis by activating the AKT signaling pathway, upregulating the p-eNOS and initiating downstream NO activation 53. Our in vitro results revealed notably elevated levels in VEGF, HIF-1α genes in rBMSCs cultured in the MBG-GO scaffold extracts compared with the purely MBG extract, which may result from relatively low concentrations of GO in the scaffold. In line with these results, areas with more new vessels were found in the MBG-GO groups in vivo via Microfil and CD34 staining evaluations. Furthermore, it is an interesting result that GO content has played a vital role in both angiogenesis and osteogenesis. The highest levels of ALP, COL1 and VEGF gene expressions were found in MBG-HGO group in vitro, and MBG-HGO can also promote osteogenesis and angiogenesis better in vivo.

In our in vivo experiment, it was found that part of the MBG had degraded after 12 weeks via van Gieson staining assay, and GO had no significant effect on the degradation of MBG. The recent studies had revealed that GO could be degraded by myeloperoxidase secreted by activated neutrophils 54, 55. However, our in vivo results showed that GO did not degrade completely after 12 weeks (Fig. 8E). Further studies are needed on the mechanism of osteogenesis-angiogensis and degradation of GO in vivo.

## Conclusions

In summary, MBG-GO scaffolds have been successfully fabricated by a high temperature calcination technique. The results showed that MBG-GO scaffolds possessed ordered macropores, as well as exhibiting good biocompatibility and stimulating proliferation of rBMSCs and osteogenic differentiation. In a bone defect model, MBG-GO scaffolds significantly enhance new bone and vessel formation in both the inner and peripheral scaffold areas in defects without the presence of growth factors or stem cells. Therefore, MBG-GO scaffolds demonstrated excellent osteogenic-angiogenic properties and will be appealing candidates for bone defect repair.

## Figures and Tables

**Fig 1:**
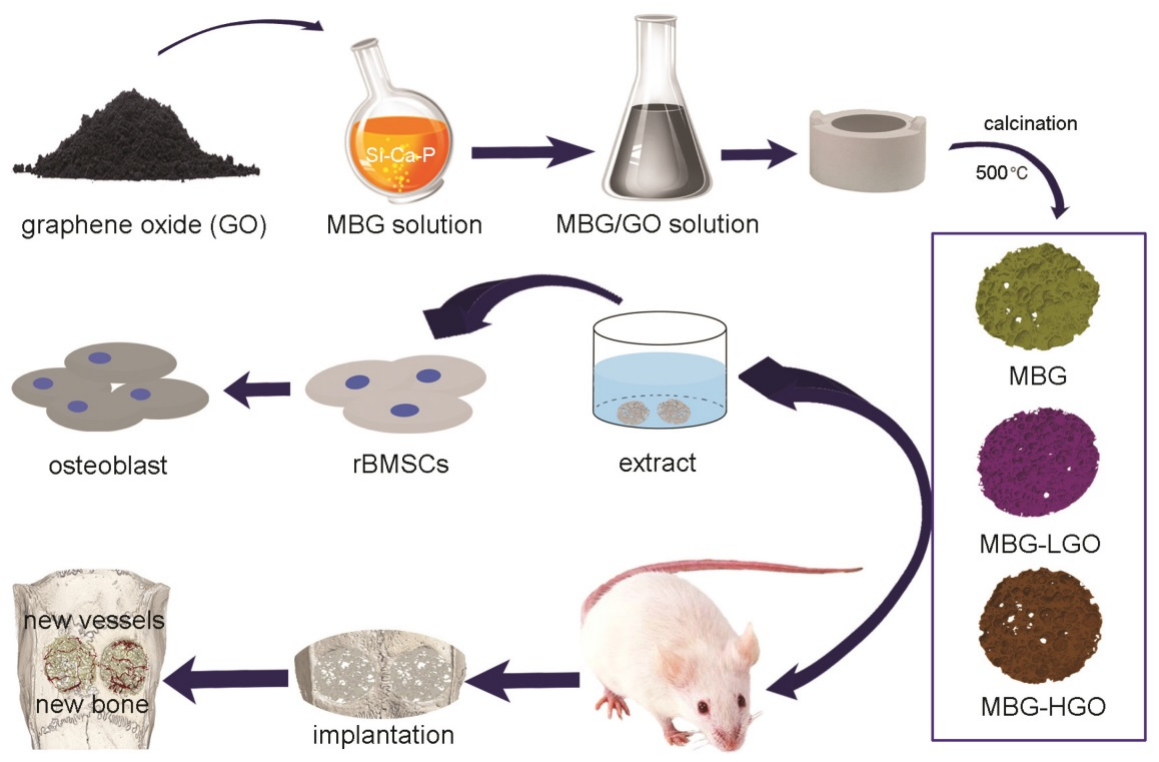
Schematic diagram of application of MBG-GO scaffolds for bone repair. The MBG-GO scaffolds were successfully synthesized by high temperature calcination method. The extract of scaffolds not only promoted cells proliferation, but also stimulated osteogenic differentiation in vitro; on the other hand, MBG-GO scaffolds significantly accelerated bone regeneration as well as promoted the formation of neovascularization in vivo.

**Table 1:** Primers used in the qRT-PCR of rBMSC cells.

Gene	Primers Sequence (F, forward; R, reverse; 5'-3')	Product Size (bp)
ALP	F: GGATCAAAGCAGCATCTTACCAG R: GCTTTCCCATCTTCCGACACT	88
COL1	F: AGAGGCATAAAGGGTCATCGTG R: AGACCGTTGAGTCCATCTTTGC	161
RUNX2	F: CCTGAACTCAGCACCAAGTCCT R: TCAGAGGTGGCAGTGTCATCA	237
OCN	F: CAGACAAGTCCCACACAGCA R: CCAGCAGAGTGAGCAGAGAG	85
HIF-1α	F: ACCGTGCCCCTACTATGTCG R: GCCTTGTATGGGAGCATTAACTT	197
VEGF	F: GAGCAGAAAGCCCATGAAGTG R: ACTCCAGGGCTTCATCATTGC	181

## Abbreviations

GO: graphene oxide
MBG: mesoporous bioactive glass
VEGF: vascular endothelial growth factor
BMP: bone morphogenetic protein
TCP: tricalcium phosphate
PLGA: poly(lactic-co-glycolic acid)
P123: EO_20_PO_70_EO_20_
TEOS: Tetraethyl orthosilicate
TEP: triethyl phosphate
FTIR: fourier transformation infrared spectrum
rBMSCs: rat bone marrow mesenchymal stem cells
SEM: scanning electron microscopy
CCK-8: Cell Counting Kit-8
ALP: alkaline phosphatase
RUNX-2: runt-related transcription factor 2
OCN: osteocalcin
COL1: collagen type 1
HIF-1α: hypoxia-inducible factor 1-alpha
qRT-PCR: quantitative reverse transcription polymerase chain reaction
Micro-CT: microcomputed tomography
CLSM: confocal laser scanning microscopy
IHC: immunohistochemical
EDTA: ethylenediaminetetraacetic acid
DMOG: dimethyloxaloylglycine
